# Decreased cardiac mortality with nicorandil in patients with ischemic heart failure

**DOI:** 10.1186/s12872-017-0577-3

**Published:** 2017-05-31

**Authors:** Akiomi Yoshihisa, Yu Sato, Shunsuke Watanabe, Tetsuro Yokokawa, Takamasa Sato, Satoshi Suzuki, Masayoshi Oikawa, Atsushi Kobayashi, Yasuchika Takeishi

**Affiliations:** Department of Cardiovascular Medicine, Fukushima Medical University, 1 Hikarigaoka, Fukushima, 960-1295 Japan

**Keywords:** Ischemic heart failure, Nicorandil, Cardiac mortality, Prognosis

## Abstract

**Background:**

Effective treatments in heart failure (HF) patients with ischemic etiology have not been fully established. Nicorandil, combination of nitrate component and sarcolemmal adenosine triphosphate-sensitive potassium channel opener, is a potent vasodilator of coronary and peripheral vessels and has been used as an antianginal agent. Therefore, we examined impacts of nicorandil on cardiac mortality in ischemic HF patients.

**Methods:**

Consecutive 334 HF patients with ischemic etiology were retrospectively registered and divided into 2 groups based on oral administration of nicorandil: nicorandil group (*n* = 116) and non-nicorandil group (*n* = 218). We retrospectively examined cardiac mortality.

**Results:**

In the Kaplan-Meier analysis (mean follow-up period 963 days), cardiac mortality was significantly lower in the nicorandil group than in the non-nicorandil group (11.2% vs. 19.7%, *P* = 0.032). In the Cox proportional hazard analysis, usage of nicorandil was a suppressor of cardiac mortality (hazard ratio 0.512, 95% confidence interval 0.275–0.953, *P* = 0.035), and this result was consistent in several subgroup analyses, such as left ventricular ejection fraction, percutaneous coronary intervention, coronary artery bypass graft, diabetes, β-blockers, and statins.

**Conclusion:**

Nicorandil is potentially effective for reducing mortality in patients with ischemic heart failure.

**Trial registration:**

This was a retrospective study.

## Background

Recent standard pharmacotherapy for heart failure (HF), such as beta-blockers and renin angiotensin system inhibitors, have much improved mortality in HF patients [1–3]. HF with ischemic etiology accounts for more than 50% of HF cases in Europe and North America, as well as 30–40% of HF cases in East Asia, and Latin America and the Caribbean [4]. Ischemic HF is associated with shorter survival than non-ischemic HF [5]. Percutaneous coronary intervention and mitral valve repair, except for coronary artery bypass graft (CABG), do not sufficiently improve the cardiac mortality rate in ischemic HF patients [6–9]. It has been recently reported that CABG added to pharmacotherapy decreases cardiovascular mortality as 10-year outcome [10]. A more comprehensive approach is necessary to refocus preventive and therapeutic strategies, and to decrease ischemic HF morbidity and mortality. Nicorandil, a combination of nitrate components and sarcolemmal adenosine triphosphate-sensitive potassium channel opener, is a potent vasodilator of coronary and peripheral vessels and has been used as an antianginal agent [11]. A recent meta-analysis revealed that nicorandil treatment in patients with ischemic heart disease did not reduce revascularization (relative risk, RR 0.95, 95% CI 0.70–1.29) or all-cause mortality (RR 0.81, 95% CI 0.64–1.02), but did reduce cardiovascular events (RR 0.77, 95% CI 0.69–0.86) [11]. Therefore, we examined the impacts of oral administration of nicorandil on cardiac mortality in ischemic HF patients.

## Methods

### Subjects and study protocol

This was a retrospective study. Consecutive 334 HF patients with ischemic etiology at Fukushima Medical University between 2009 and 2014 were divided into two groups based on oral administration of nicorandil at hospital discharge: a nicorandil group (guideline-based medical therapy + nicorandil 5 mg tid, *n* = 116) and non-nicorandil group (guideline-based medical therapy alone, *n* = 218). While the prescription of nicorandil was determined by the attending physician freely, patients with advanced coronary artery disease tended to be prescribed nicorandil in our hospital. Diagnosis of decompensated HF was defined based on the Framingham criteria [12]. Ischemic etiology was confirmed by either myocardial scintigraphy or coronary computed tomography angiography and/or coronary angiography. The study protocol conforms to the ethical guidelines of the 1975 Declaration of Helsinki as reflected in a prior approval by the institution’s human research committee. We compared clinical features between the two groups. All patients were followed up for cardiac death until 2016. Cardiac death was adjudicated by independent experienced cardiologists and included death due to worsened HF in accordance with the Framingham criteria [12], ventricular fibrillation documented by electrocardiogram or other implantable devices, and acute coronary syndrome.

### Statistical analysis

The chi-square test was used for comparisons of categorical variables. Data of the two groups were compared using the independent Student’s t-test for normally distributed data, and the Mann-Whitney U test for non-normally distributed data. To assess the potential heterogeneity of nicorandil treatment effects on cardiac mortality, we conducted subgroup analyses. Interactions between nicorandil and the following clinically relevant variables, which are different between the two groups and/or generally known risk factors, were estimated by a Cox proportional hazards regression model: age, sex, left ventricular ejection fraction (LVEF), presence of left main trunk lesion, three-vessel disease, history of percutaneous coronary intervention or CABG, presence of diabetes, chronic kidney disease, dialysis, and use of β-blockers, statins, anti-platelet agents, and nitrate. A value of *P* < 0.05 was considered statistically significant for all comparisons. Analyses were performed using a statistical software package (SPSS ver. 21.0, IBM, Armonk, NY, USA).

## Results

**Fig. 1:**
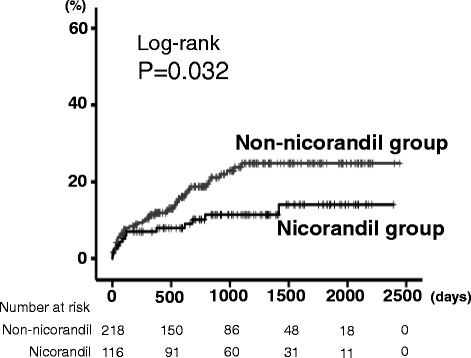
Comparison of cardiac mortality between the nicorandil (*n* = 116) and non-nicorandil groups (*n* = 218)

**Fig. 2:**
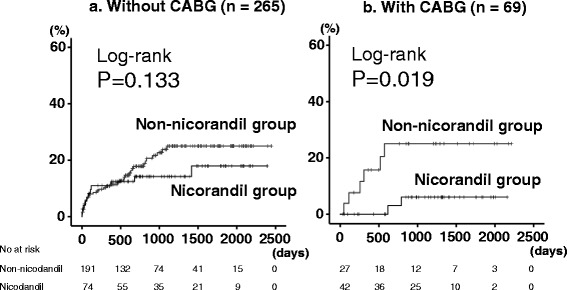
Comparison of cardiac mortality between the nicorandil and non-nicorandil groups in patients with or without coronary artery bypass graft (CABG): **a**. Without CABG (*n* = 265) and **b**. With CABG (*n* = 69)

As shown in Table 1, the nicorandil group had higher prevalence of three-vessel disease, history of coronary artery bypass graft, usage of anti-platelet agents and statins, and tended to have higher prevalence of diabetes and usage of β-blockers and nitrates. In contrast, age, gender, New York Heart Association class, other co-morbidities, B-type natriuretic peptide, C-reactive protein, total protein, sodium, and LVEF did not differ between the two groups. During the follow-up period (mean 963 days), there were 56 cardiac deaths (13 in the nicorandil group and 43 in the non-nicorandil group). As shown in Fig. 1, the cardiac mortality was significantly lower in the nicorandil group than in the non-nicorandil group (*P* = 0.032). In the Cox proportional hazard analysis (Table 2), usage of nicorandil was a suppressor of cardiac mortality (HR 0.512, 95%CI 0.275–0.953, *P* = 0.035). Interactions between nicorandil use and clinically relevant variables were modeled using Cox regression and are shown in Table 2 for cardiac mortality. In the subgroup analysis, there was no interaction between nicorandil use and other important variables that affect cardiac mortality in any subgroups. Then, we focused on the history of CABG (Fig. 2), cardiac mortality was significantly lower in the nicorandil group than in the non-nicorandil group in patients with CABG (*P* = 0.019), and remained in a tendency in patients without CABG (*P* = 0.133).

**Table 1:** Comparisons of clinical features (*N* = 334)

	Non-nicorandil group (*n* = 218)	Nicorandil group (*n* = 116)	*P*-value
Age (years)	71.7 ± 11.6	69.8 ± 10.5	0.146
Male gender (*n*, %)	169 (77.5)	86 (74.1)	0.488
Body mass index (kg/cm^2^)	23.7 ± 4.6	23.9 ± 4.2	0.708
Systolic blood pressure (mmHg)	132.3 ± 36.1	130.8 ± 35.9	0.716
Diastolic blood pressure (mmHg)	76.2 ± 23.3	72.4 ± 21.5	0.144
Heart rate (bpm)	82.2 ± 23.8	77.5 ± 21.2	0.071
New York Heart Association class III or IV (*n*, %)	5 (2.3)	3 (2.6)	0.868
LVEF (%)	43.3 ± 13.6	45.6 ± 14.5	0.211
LMT lesion (*n*, %)	9 (4.1)	10 (8.6)	0.134
3VD (*n*, %)	45 (20.6)	40 (34.5)	0.008
PCI (*n*, %)	159 (72.9)	86 (74.1)	0.813
CABG (*n*, %)	27 (12.4)	42 (36.2)	<0.001
Co-morbidity			
Hypertension (*n*, %)	194 (89.0)	105 (90.5)	0.665
Diabetes (*n*, %)	128 (58.7)	79 (68.1)	0.092
Dyslipidemia (*n*, %)	193 (88.5)	107 (92.2)	0.286
Atrial fibrillation (*n*, %)	64 (29.4)	27 (23.3)	0.235
Chronic kidney disease (*n*, %)	151 (69.3)	78 (67.2)	0.704
Dialysis (*n*, %)	28 (12.8)	16 (13.8)	0.865
Anemia (*n*, %)	141 (64.7)	78 (67.2)	0.639
Smoking (*n*, %)	155 (71.1)	74 (63.8)	0.171
Medications			
Angiotensin converting enzyme inhibitors (*n*, %)	123 (56.4)	71 (61.2)	0.417
Angiotensin receptor blockers (*n*, %)	67 (30.7)	36 (31.0)	1.000
Aldosterone antagonists (*n*, %)	91 (41.7)	42 (36.2)	0.325
β-blockers (*n*, %)	176 (80.7)	102 (87.9)	0.094
Calcium channel blockers (*n*, %)	94 (43.1)	47 (40.5)	0.727
Diuretics (*n*, %)	153 (70.2)	79 (68.1)	0.694
Inotropic agents (*n*, %)	27 (12.4)	11 (9.5)	0.426
Anti-platelet agents (*n*, %)	186 (85.3)	113 (97.4)	<0.001
Anti-coagulations (*n*, %)	97 (44.5)	50 (43.1)	0.807
Anti-diabetic agents (*n*, %)	92 (42.2)	59 (50.9)	0.135
Statins (*n*, %)	132 (60.6)	88 (75.9)	0.005
Nitrates (*n*, %)	44 (20.2)	34 (29.3)	0.077
Laboratory data			
BNP (pg/ml)^a^	306.5 (865.1)	377.5 (619.8)	0.374
C-reactive protein (mg/dl)^a^	0.32 (1.19)	0.21 (0.78)	0.132
Total protein (g/dl)	7.0 ± 0.8	7.0 ± 0.7	0.816
Sodium (mEq/l)	138.2 ± 4.2	138.6 ± 3.5	0.492

*LVEF* left ventricular ejection fraction, *LMT* left main trunk, *3VD* three-vessel disease, *PCI* percutaneous coronary intervention, *CABG* coronary artery bypass graft, *BNP* B-type natriuretic peptide

^a^Data are presented as median (interquartile range)

**Table 2:** Subgroup analysis for cardiac mortality: Nicorandil use

Factor	Subgroup	n	HR	95% Cl	*P* value	Interaction *P* value
Total		334	0.512	0.275–0.953	0.035	-
Age	≥75	143	0.807	0.344–1.890	0.621	0.252
	<75	191	0.380	0.153–0.942	0.037	
Sex	Male	255	0.449	0.216–0.932	0.032	0.403
	Female	79	0.737	0.213–2.547	0.629	
LVEF	Reduced	244	0.623	0.325–1.192	0.153	0.405
	Preserved	90	0.245	0.029–2.102	0.200	
LMT	Present	19	1.240	0.000–3.420	0.581	0.968
	Absent	315	0.492	0.259–0.934	0.030	
3VD	Present	85	0.672	0.244–1.849	0.441	0.482
	Absent	249	0.425	0.188–0.962	0.040	
PCI	Present	245	0.556	0.272–1.138	0.108	0.646
	Absent	89	0.422	0.120–1.483	0.179	
CABG	Present	69	0.181	0.036–0.897	0.036	0.128
	Absent	265	0.718	0.366–1.409	0.336	
Diabetes	Present	207	0.412	0.177–0.957	0.039	0.361
	Absent	127	0.742	0.296–1.858	0.523	
CKD	Present	229	0.434	0.217–0.871	0.019	0.206
	Absent	105	1.252	0.280–5.596	0.769	
Dialysis	Present	44	0.338	0.073–1.568	0.166	0.595
	Absent	290	0.557	0.282–1.100	0.092	
β-blockers	Present	278	0.483	0.229–1.022	0.057	0.469
	Absent	56	0.830	0.273–2.523	0.743	
Statins	Present	220	0.720	0.324–1.604	0.422	0.425
	Absent	114	0.400	0.139–1.153	0.090	
Anti-platelet agents	Present	299	0.600	0.316–1.140	0.119	0.907
	Absent	35	0.041	0.000–215.058	0.464	
Nitrates	Present	78	0.551	0.188–1.616	0.277	0.814
	Absent	256	0.474	0.219–1.027	0.058	

*LVEF* left ventricular ejection fraction, *LMT* left main trunk, *3VD* three-vessel disease, *PCI* percutaneous coronary intervention, *CABG* coronary artery bypass graft, *CKD* chronic kidney disease

## Discussion

In the present study, we firstly demonstrated that oral administration of nicorandil was associated with lower cardiac mortality in ischemic HF patients, and this result was consistent in several subgroup analyses, such as LVEF, percutaneous coronary intervention, coronary artery bypass graft, diabetes, β-blockers, and statins.

Intravenous nicorandil for decompensated HF patients, regardless of ischemic etiology, improves cardiac pump function, New York Heart Association class, left ventricular function, myocardial microvascular circulation, pulmonary capillary wedge pressure, pulmonary arterial pressure, and peripheral resistance [13], and oral administration of nicorandil decreases the composite end point of mortality and hospitalization for cardiac causes (HR 0.35, 95% CI 0.16–0.54) [13]. Oral administration of nicorandil suppresses sympathetic nervous activity, prevents left ventricular remodeling in HF patients (LVEF <45%, ischemic etiology 43.5%), and may reduce cardiac events (cardiac mortality, HR 0.502, 95% CI 0.268–0.940; major adverse cardiac effect, HR 0.436, 95% CI 0.266–0.715) [14]. These previous reports [13, 14] are partially concordant with our results.

Several favorable effects of nicorandil on cardiovascular system have been reported, such as reduction in preload and afterload, improvement of myocardial perfusion, protection of cardiomyocytes from ischemic damage, prevention of Ca^2+^ overload by opening adenosine triphosphate-sensitive potassium channels, anti-inflammatory and anti-proliferative effects, anti-apoptosis, anti-arrhythmic effects, protection of endothelial, mitochondrial, and energy-modulating functions, and preservation of kidney function [11, 13, 14].

### Study limitations

There are several limitations in the present study. First, it is a nonrandomized and retrospective study of a single institution, so the number of subjects was relatively small and there are potential biases and confounders that may be responsible for our findings. Second, we have conducted this study using only variables on hospitalization, without consideration for changes in medical parameters and post-discharge treatment. Third, our results has not established a cause-effect relationship between the usage of nicorandil and improvement of cardiac mortality. Thus, the results of the present study should be viewed as preliminary, and further studies with larger populations and randomization are needed.

## Conclusions

In conclusion, nicorandil potentially reduces cardiovascular mortality in patients with ischemic HF.

## Abbreviations

CABG: Coronary artery bypass graft
CI: Confidence interval
HF: Heart failure
HR: Hazard ratio
LVEF: Left ventricular ejection fraction
RR: Relative risk
